# Communication needs regarding heart failure trajectory and palliative care between patients and healthcare providers: A cross-sectional study

**DOI:** 10.1371/journal.pone.0317417

**Published:** 2025-01-13

**Authors:** Jin-Sun Park, Kyoung-Woo Seo, Jung Eun Lee, Kyoung-Hwa Kim, Jeong-Ah Ahn

**Affiliations:** 1 Department of Cardiology, Ajou University School of Medicine, Suwon, Korea; 2 College of Nursing, University of Rhode Island, Kingston, RI, United States of America; 3 College of Nursing and Research Institute of Nursing Science, Ajou University, Suwon, Korea; Uniwersytet Jagiellonski w Krakowie Biblioteka Jagiellonska, POLAND

## Abstract

**Introduction:**

Heart failure (HF) is a chronic condition with an unpredictable trajectory, making effective communication between patients and healthcare providers crucial for optimizing outcomes. This study aims to investigate and compare the communication needs regarding HF trajectory and palliative care between patients and healthcare providers and to identify factors associated with the communication needs of patients with HF.

**Methods:**

A cross-sectional study design was employed, involving 100 patients with HF and 35 healthcare providers. Data were collected using structured questionnaires assessing communication needs, health literacy, self-care behavior, and social support. Statistical analyses were performed, including Spearman’s rank correlation, Pearson’s correlation, and multiple regression analyses.

**Results:**

Patients prioritized communication related to device-related questions, whereas healthcare providers focused more on aspects of HF in daily life. Both groups ranked end-of-life communication as the lowest priority. The communication needs of patients were positively correlated with health literacy (r = 0.27, *p* = .007), self-care behavior (r = 0.32, *p* = .001), and social support (r = 0.24, *p* = .016). Multiple regression analyses indicated that self-care behavior was a significant factor influencing the communication needs of patients (β = 0.27, *p* = .011).

**Conclusions:**

Enhanced patient-centered communication strategies are required to address the communication priority gaps between patients and healthcare providers. Improving health literacy, supporting self-care behaviors, and leveraging social support are critical in meeting patients’ communication needs. Tailored communication training for healthcare providers can bridge this gap and improve overall HF management.

## Introduction

The pathogenesis of cardiovascular disease is diverse, and even after proper initial treatment, most patients progress from chronic heart disease to heart failure (HF), which inflicts considerable morbidity and mortality [1]. HF remains a chronic disease with steadily increasing burdens of hospitalization and healthcare costs [2]. Moreover, in recent years, the incidence of cardiovascular diseases has been escalating due to the aging population, which naturally leads to an increase in the number of patients with HF [3]. In a recent review, HF has been described as a global pandemic since it affects more than 64 million people worldwide [4]. Not only is the prevalence of HF increasing significantly, but the hospitalization rate is also rising. HF is reported to be the most common cause of hospitalization and readmission for patients aged 65 years and older, with a readmission rate of approximately 20% for all patients with HF and a readmission rate of approximately 70% within 3 months of discharge, resulting in a heavy healthcare cost burden for patients, their families, and the healthcare system [5]. Despite major therapeutic advances in recent decades, HF is a chronic disease that is difficult to cure, and patients and their families can experience uncertainty, stress, anxiety, and burden, leading to a decrease in overall quality of life [6]. This, in turn, adversely affects the patient’s lifelong self-management of their HF, which is an essential component of overall HF care [2, 7].

Guidelines for the management of HF recommend that the primary goals are to improve prognosis and mortality, alleviate symptoms, and enhance quality of life [8, 9]. This requires a coordinated effort that actively involves not only healthcare providers but also patients and families in decision-making about the direction of care plans based on their preferences [10]. Therefore, effective communication strategies between healthcare providers, patients, and families, in accordance with the disease trajectory of HF, should be established [11, 12]. Because the disease trajectory of HF is variable and unpredictable, these communications must include personalized and sufficient explanations by healthcare providers and a commitment to the patient’s understanding. A lack of understanding of the disease trajectory and end-of-life issues in patients with HF, combined with sensitive dialogue, can increase patient anxiety and undermine treatment plans, negatively impacting patient outcomes [13, 14]. Especially for end-of-life palliative care, proactive communication is necessary to ensure appropriate healthcare utilization and provide proper end-of-life care to patients and their families [15].

The importance of communication in the evolving disease trajectory of HF, as well as palliative and supportive care, has been emphasized in recent studies [16–19]. It is recommended to provide palliative and supportive care to patients with HF, including prognosis communication, clarification of treatment goals, shared decision-making, symptom management, and caregiver support, to alleviate symptoms and improve quality of life [14, 20]. Patient-centered care and empowerment are foundational to improving communication in HF management. Patient-centered care focuses on tailoring care to the unique needs, values, and preferences of patients, fostering collaboration among patients, caregivers, and healthcare providers [21]. Also, empowerment complements this by equipping both patients and caregivers with the skills, knowledge, and confidence needed to actively engage in care decisions [22]. Active communication that incorporates these aspects of patient-centered care and empowerment plays an essential role in enhancing self-care involvement for patients and their families, while reducing anxiety, stress, and burden during disease progression and at the end of life [23].

However, current communication between healthcare providers and patients is often reported to be limited, primarily focusing on medical treatments for physical conditions rather than addressing broader concerns such as the psychosocial aspects of care, disease trajectory, and end-of-life planning [24]. This gap is compounded by the fact that many healthcare providers lack the necessary training to effectively communicate these sensitive and complex issues [25]. As a result, critical conversations that could enhance patient understanding, alleviate anxiety, and guide decision-making are frequently inadequate or absent [26]. Additionally, many patients face significant challenges in communicating with healthcare providers, often due to a lack of tailored communication strategies that account for their specific needs and unique circumstances, including literacy levels, self-care skills, and social support [25, 27, 28]. These difficulties can lead to misunderstandings, increased anxiety, and suboptimal care [29]. Identifying the factors associated with patients’ communication needs is therefore crucial, and there is an urgent need for research aimed at improving communication in HF care to ensure that both patients’ and providers’ needs are met comprehensively.

Therefore, this study aims to investigate the communication needs and associated factors of patients and healthcare providers regarding HF trajectory and palliative care as a basis for future strategy development.

## Materials and methods

### Study design

The present investigation was a cross-sectional, descriptive, and exploratory study.

### Setting and samples

One hundred patients with HF and 35 healthcare providers (30 nurses and 5 physicians) were recruited from the outpatient cardiovascular department of a large tertiary medical center in South Korea, considering a population actively managing chronic HF in a clinical setting. Data were collected from November 9, 2022 to March 30, 2023. Patients were eligible if they were adults aged 19 years or older, had been diagnosed with HF by a cardiologist, and were receiving ongoing care through regular medical follow-ups, reflecting the typical demographic of patients with HF under continuous management. Healthcare providers were eligible if they were nurses or physicians with more than 5 years of clinical experience in the cardiovascular department of the medical center, ensuring their expertise and familiarity with the patients with HF.

### Measurements

#### Communication needs about heart failure trajectory and palliative care

Communication needs were measured using the Question Prompt List (QPL) for communication about the HF trajectory and palliative care, developed by Hjelmfors et al. [30]. Permission to use the English version of the tool was obtained from the original authors. The Korean version was developed in accordance with the suggested guidelines [31]. First, a bilingual nursing professor among the authors performed a forward translation of the original tool from English to Korean. Next, a professional bilingual translator performed a backward translation from Korean to English. After reviewing the back-translated version for linguistic consistency with a cardiologist and a cardiac nurse, it was sent to the original authors, who approved the final Korean version of the tool. The QPL for communication about the HF trajectory and palliative care consists of 33 questions across 5 sections: HF in daily life, support when deteriorating, end-of-life, questions for family members, and device-related questions. The original version was answered using yes/no/do not know options. However, this study used a 5-point Likert scale (0 = not necessary at all; 4 = very necessary) to determine the level of communication needs. The Korean version of the tool demonstrated a reliability (Cronbach’s α) of .92 in the present study.

#### Health literacy

Health literacy was measured using the Korean version of the HF-Specific Health Literacy Scale [32], originally developed by Matsuoka et al. [33]. This 12-item tool comprises three domains: functional, communicative, and critical literacy. Each item is rated on a 4-point Likert scale (1 = strongly disagree; 4 = strongly agree), and the total score ranges from 12 to 48, with higher scores indicating higher health literacy. The HF-Specific Health Literacy Scale demonstrated a reliability (Cronbach’s α) of .70 in the present study.

#### Self-care behavior

Self-care behavior was assessed using the Korean version of the 9-item European HF Self-care Behavior Scale (EHFScBS-9) [34], originally developed by Jaarsma et al. [35]. This tool comprises 9 items. Each item is rated on a 5-point Likert scale (1 = completely agree; 5 = completely disagree), and the scores are converted to a standardized 100-point scale (0–100) using the suggested formula [36], with higher scores indicating better self-care. The self-care behavior measurement demonstrated a reliability (Cronbach’s α) of .73 in the present study.

#### Social support

Social support was measured using the Korean version of the Multidimensional Scale of Perceived Social Support [37], originally developed by Zimet et al. [38]. This tool consists of 12 items in three domains: support from significant others (e.g., healthcare providers), from family, and from friends. Each item is rated on a 5-point Likert scale (1 = strongly disagree; 5 = strongly agree), with the total score ranging from 12 to 60. Higher scores indicate higher perceived social support. The Multidimensional Scale of Perceived Social Support demonstrated a reliability (Cronbach’s α) of .73 in the present study.

### Ethical considerations

This study was approved by the Institutional Review Board of Ajou University Medical Center (IRB no. AJOUIRB-SB-2022-420). Written informed consent was obtained from all participants who volunteered to take part in the study. Participants were informed that their involvement was entirely voluntary and that they could refuse participation or withdraw their consent at any time without facing any disadvantages. In addition, it was explained that all collected data would remain confidential.

### Data analyses

Data collected in this study were analyzed using SPSS version 25.0 (IBM Corporation, Armonk, NY, USA). Descriptive statistics were used to summarize participants’ characteristics. Mean scores for communication needs were ranked from highest to lowest within each participant group. Independent t-tests and Spearman’s rank correlation tests were used to identify differences and relationships between the communication needs reported by patients and healthcare providers. The correlations between patients’ characteristics and their communication needs were presented using Pearson’s correlation coefficients. Lastly, the factors associated with the communication needs of patients were analyzed using multiple regression analysis.

## Results

### Characteristics of participants

Table 1 presents the characteristics of the participants. The mean age of 100 patients with HF was 64.62 years, with 66.0% being men. Among the patients, 65.0% were married, 74.0% had a high school education or below, and 57.0% were currently unemployed. The average duration of HF diagnosis was 6.40 years. The mean scores for health literacy, self-care behavior, and social support were 32.30/48, 61.94/100, and 44.42/60, respectively.

**Table 1:** Characteristics of patients with heart failure and healthcare providers.

Characteristics	Categories	Patients (n = 100)	Healthcare providers (n = 35)
		n (%) or *Mean±SD*	
Age (year)		*64*.*62±14*.*30*	*34*.*76±7*.*05*
Gender	Men	66 (66.0)	11 (31.4)
	Women	34 (34.0)	24 (68.6)
Marital status	Unmarried	13 (13.0)	19 (54.3)
	Married	65 (65.0)	16(45.7)
	Divorced	9 (9.0)	0 (0.0)
	Bereaved	13 (13.0)	0 (0.0)
Education level	High school and below	74 (74.0)	0 (0.0)
	College/university	26 (26.0)	22 (62.9)
	Master/doctoral degree	0 (0.0)	13 (37.1)
Working status	Yes	43 (43.0)	35 (100.0)
	No	57 (57.0)	0 (0.0)
Clinical career (year)		*-*	*10*.*90±6*.*94*
Duration of heart failure (year)		*6*.*40±5*.*41*	-
Health literacy (12–48)		*32*.*30±6*.*56*	-
Self-care behavior (0–100)		*61*.*94±19*.*31*	-
Social support (12–60)		*44*.*42±9*.*32*	-

Abbreviations: SD, standard deviation.

The mean age of the 35 healthcare providers was 34.76 years, with 68.6% being women. Of these providers, 62.9% had graduated from college or university, and 37.1% had a master’s or doctoral degree. The average length of clinical experience in cardiology for the healthcare providers was 10.90 years.

### Comparison of communication needs about disease trajectory and palliative care between patients and healthcare providers

The total mean scores for communication needs of patients and healthcare providers were 3.15 and 3.05 out of 4, respectively. “Device-related questions” was the highest priority communication area for patients (mean = 3.90), while “HF in daily life” was the highest priority for healthcare providers (mean = 3.35). On the other hand, “End-of-life” was the lowest priority for both patients (mean = 3.12) and healthcare providers (mean = 2.80).

**Fig 1:**
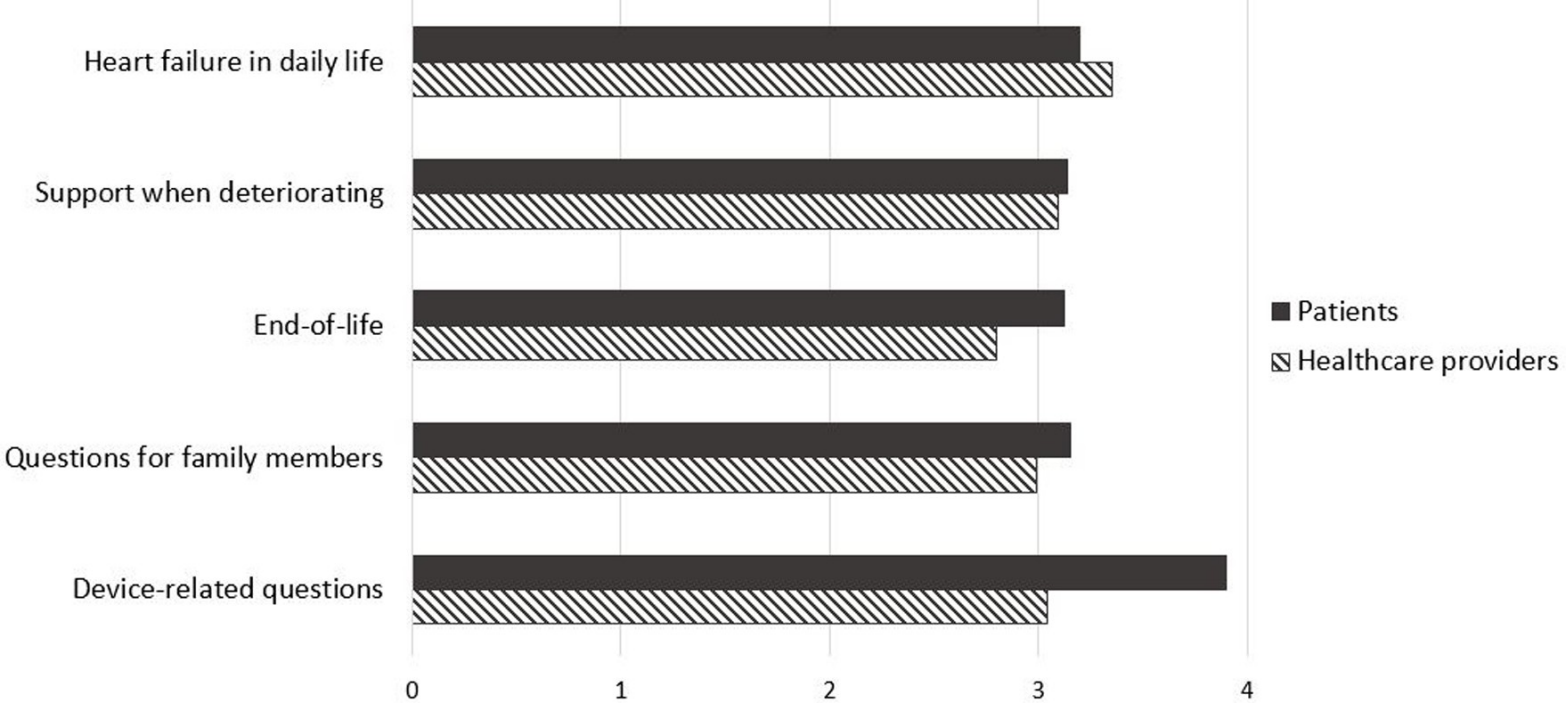
Comparison of communication needs about disease trajectory and palliative care between patients with heart failure and healthcare providers according to communication topics.

When comparing the mean scores for communication needs between patients and healthcare providers, no significant differences were identified. However, the rank correlation between the communication needs of the two groups was 0.32 (*p* < .05 by Spearman test), indicating that the rank orders for communication needs were significantly different (Table 2 and Fig 1).

**Table 2:** Comparison of communication needs about disease trajectory and palliative care between patients with heart failure and healthcare providers.

Communication needs	Patients (n = 100)		Healthcare providers (n = 35)		t (*p*)
	Mean±SD	Rank	Mean±SD	Rank	
Heart failure in daily life	3.20±0.90	2	3.35±0.44	1	-1.29 (.201)
Support when deteriorating	3.14±0.93	4	3.09±0.50	2	0.39 (.694)
End-of-life	3.12±1.06	5	2.80±0.77	5	1.89 (.062)
Questions for family members	3.15±0.98	3	2.99±0.49	4	1.21 (.230)
Device-related questions	3.90±0.02	1	3.04±0.61	3	1.56 (.128)
Total	3.15±0.77		3.05±0.42		0.96 (.340)

Abbreviations: SD, standard deviation.

Ranks are presented from the highest to the lowest score.

### Associated factors of patients’ communication needs about disease trajectory and palliative care

The total mean score for communication needs of the patients showed significantly positive correlations with health literacy (r = 0.27, *p* = .007), self-care behavior (r = 0.32, *p* = .001), and social support (r = 0.24, *p* = .016) (Table 3).

**Table 3:** Correlations between patients’ characteristics and communication needs about disease trajectory and palliative care (*N* = 100).

Characteristics	1	2	3	4	5	6
	r (*p*)					
1. Age	1.00					
2. Duration of heart failure	0.06 (.589)	1.00				
3. Health literacy	0.08 (.417)	0.04 (.678)	1.00			
4. Self-care behavior	0.31 (.002)	0.08 (.444)	0.43 (< .001)	1.00		
5. Social support	0.03 (.749)	-0.07 (.492)	0.26 (.010)	0.14 (.158)	1.00	
6. Communication needs	0.17 (.084)	0.03 (.790)	0.27 (.007)	0.32 (.001)	0.24 (.016)	1.00

Additionally, a multiple regression model showed that patients’ self-care behavior was a significant factor associated with communication needs (F = 5.94, *p* = .001, adj. R^2^ = .131). That is, communication needs were significantly higher as patients’ self-care behaviors increased (β = 0.27, *p* = .011) (Table 4).

**Table 4:** Associated factors of communication needs about disease trajectory and palliative care of patients with heart failure (*N* = 100).

Factors	B	SE	β	t	*p*
(constant)	2.86	0.62		4.63	< .001
Health literacy	0.01	0.01	0.09	0.85	.396
Self-care behavior	0.03	0.01	0.27	2.60	.011
Social support	0.01	0.01	0.18	1.84	.069
R^2^ = .158, adjusted R^2^ = .131, F = 5.94, *p* = .001					

Abbreviations: SE, standard error.

## Discussion

This study provides an in-depth understanding of the communication needs between patients and healthcare providers regarding HF trajectory and palliative care. As one of the first attempts to compare aspects of communication between patients and healthcare providers regarding HF, the findings provide important insights. They highlight the need for communication strategies that consider the differences in communication priorities between patients and healthcare providers, as well as the significant role of patients’ characteristics in shaping their communication needs.

This study’s results clearly highlight substantial differences in communication needs between patients with HF and healthcare providers. Patients expressed a greater need for information regarding the care options related to the progression of their disease, particularly regarding “device-related questions”. In contrast, healthcare providers focused primarily on the medical treatment aspects, such as “HF in daily life”, addressing this gap is crucial. To enhance patient-centered care, it is essential to provide more comprehensive communication on questions related to care device options, such as implantable cardioverter defibrillators or HF pacemakers [39]. Doing so can alleviate patient anxiety, support informed decision-making, and ultimately improve treatment adherence and health outcomes.

The lower prioritization of end-of-life discussions by both patients and healthcare providers in this study is noteworthy. This reluctance might originate from cultural sensitivities and discomfort with discussing mortality, particularly in cultures where such topics are considered taboo [40]. The avoidance can result in missed opportunities for patients to express their wishes and for healthcare providers to offer appropriate guidance and support. Additionally, healthcare providers often lack training in initiating these conversations, resulting in a greater focus on curative treatments rather than palliative care [41]. However, proactive end-of-life communication can significantly improve the quality of life for terminally ill patients with HF by aligning care with patient preferences, reducing unnecessary hospitalizations, and decreasing anxiety and depression [42, 43]. Moreover, such discussions can increase family satisfaction with care and reduce stress during bereavement [44]. Therefore, raising awareness and implementing communication training for healthcare providers are crucial for improving end-of-life care for patients with HF.

The study also identified key factors associated with patients’ communication needs. Health literacy and social support were positively correlated with higher communication needs, suggesting that patients who are more knowledgeable about their condition and those with robust support networks seek more information and engagement in their care [10, 45]. Health literacy plays a particularly significant role, underscoring the importance of educational interventions tailored to patients’ understanding levels [46]. It impacts not only the understanding of medical information but also the ability to navigate the healthcare system, engage in self-care practices, and make informed health decisions [47]. Also, patients with strong social networks are more likely to be actively involved in their care, facilitated by the encouragement and assistance of family or friends [48]. Social support can enhance patients’ confidence in managing their condition and foster more open and frequent communication with healthcare providers [49]. These findings align with previous studies that highlight the positive impact of social support on health outcomes and patient engagement in HF care [50, 51].

In addition, self-care behavior emerged as a crucial factor in this study, with higher levels of self-care behavior associated with greater communication needs. Patients who actively manage their condition tend to seek more detailed information and guidance from their healthcare providers [52]. This finding highlights the importance of supporting patients in developing effective self-care practices, which enhances their engagement in communication about HF management. The relationship observed suggests that as patients become more proficient in self-care, they encounter more complex questions, necessitating clearer and more frequent communication with their healthcare providers [53]. Healthcare providers can facilitate this by offering tailored education and encouraging patients to actively participate in their care communication. Integrating structured self-care education programs into routine HF management can empower patients, leading to improved outcomes and more effective communication [54]. This highlights the value of a comprehensive approach that considers various factors influencing patient communication needs.

The findings highlight the need for healthcare providers to tailor communication strategies to address the diverse needs of patients with HF. Healthcare providers should expand discussions beyond medical treatments to include long-term care planning, device-related questions, and end-of-life considerations, ensuring patients feel informed and secure in their HF management [14, 55]. Training programs should emphasize patient-centered communication, equipping healthcare providers with the skills to effectively discuss complex topics such as disease progression and palliative care [24, 49]. Implementing structured communication frameworks and decision aids can further enhance these conversations, making them more comprehensive and individualized to patient needs [56, 57]. It is also crucial to foster a collaborative environment where patients and their families feel empowered to express concerns. Including family members can ensure a more holistic, family-centered approach to HF management [48]. Technology, such as telemedicine platforms using websites or smartphone applications, can also enhance communication by providing continuous, personalized support, bridging the gap between in-person consultations [58].

While this study provides valuable insights, it has several limitations that must be addressed in future research. First, the sample size, particularly of healthcare providers, was relatively small, which may affect the generalizability of the findings. Additionally, recruitment occurred at one hospital in Korea, which further limits the generalizability of the results. Future studies should aim for more extensive and diverse participant groups, including family caregivers and multidisciplinary healthcare teams, to validate the results. Second, since the current cross-sectional design limits the ability to establish causality, future research should employ longitudinal designs to examine how communication needs evolve over time and during different stages of HF. Understanding these dynamics can help refine communication strategies to better support patients throughout their disease trajectory. Also, qualitative research methods could provide in-depth insights into the personal experiences and specific needs regarding communication from both patients’ and healthcare providers’ perspectives. Lastly, exploring the impact of cultural and socio-economic factors on communication needs could provide a better understanding, leading to the development of more targeted and effective communication strategies for patients with HF.

## Conclusions

This study highlighted significant differences in communication needs between patients with HF and healthcare providers. Patients prioritized device-related communication, whereas healthcare providers focused more on HF in daily life, with both groups ranking end-of-life communication as the lowest priority. Furthermore, the study identified self-care behavior as the key factor associated with patients’ communication needs, emphasizing the importance of tailored communication strategies that address these differences and support patient-centered care.
